# Expansion of targetable sites for the ribonucleoprotein-based CRISPR/Cas9 system in the silkworm *Bombyx mori*

**DOI:** 10.1186/s12896-021-00714-6

**Published:** 2021-09-20

**Authors:** Yun-long Zou, Ai-jun Ye, Shuo Liu, Wen-tao Wu, Li-feng Xu, Fang-yin Dai, Xiao-ling Tong

**Affiliations:** State Key Laboratory of Silkworm Genome Biology; Key Laboratory of Sericultural Biology and Genetic Breeding, Ministry of Agriculture and Rural Affairs; College of Sericulture, Textile and Biomass Sciences, Southwest University, Chongqing, 400715 People’s Republic of China

**Keywords:** CRISPR/Cas9, Ribonucleoprotein, Silkworm, Target repertoire, *BmBLOS2*, *BmGR66*

## Abstract

**Background:**

With the emergence of CRISPR/Cas9 technology, multiple gene editing procedures became available for the silkworm. Although binary transgene-based methods have been widely used to generate mutants, delivery of the CRISPR/Cas9 system via DNA-free ribonucleoproteins offers several advantages. However, the T7 promoter that is widely used in the ribonucleoprotein-based method for production of sgRNAs in vitro requires a 5′ GG motif for efficient initiation. The resulting transcripts bear a 5′ GG motif, which significantly constrains the number of targetable sites in the silkworm genome.

**Results:**

In this study, we used the T7 promoter to add two supernumerary G residues to the 5′ end of conventional (perfectly matched) 20-nucleotide sgRNA targeting sequences. We then asked if sgRNAs with this structure can generate mutations even if the genomic target does not contain corresponding GG residues. As expected, 5′ GG mismatches depress the mutagenic activity of sgRNAs, and a single 5′ G mismatch has a relatively minor effect. However, tests involving six sgRNAs targeting two genes show that the mismatches do not eliminate mutagenesis in vivo, and the efficiencies remain at useable levels. One sgRNA with a 5′ GG mismatch at its target performed mutagenesis more efficiently than a conventional sgRNA with 5′ matched GG residues at a second target within the same gene. Mutations generated by sgRNAs with 5′ GG mismatches are also heritable. We successfully obtained null mutants with detectable phenotypes from sib-mated mosaics after one generation.

**Conclusions:**

In summary, our method improves the utility and flexibility of the ribonucleoprotein-based CRISPR/Cas9 system in silkworm.

**Supplementary Information:**

The online version contains supplementary material available at 10.1186/s12896-021-00714-6.

## Background

In the postgenomic era, genetically modified model organisms will become essential tools as researchers turn their attention to the functional dissection of the genome. The mulberry silkworm *Bombyx mori* is not only an important lepidopteran model but is also an economically important insect in the silk industry. The genetic modification of this organism will therefore benefit basic research as well as silk production. Zinc-finger nucleases (ZFNs) and transcription activator-like effector nucleases (TALENs) have been exploited for genomic engineering in *Bombyx mori* [1, 2]*.* However, difficulties in designing and engineering ZFNs and TALENs have greatly limited the application of gene editing in silkworms. With the development of CRISPR/Cas9, gene editing in silkworms has become far more tractable [3, 4].

Gene editing using CRISPR/Cas9 in silkworms has typically been performed with a binary transgenic system [3, 5–7]. This system requires two separate transgenic lines, one of which expresses universal Cas9 and the other a customized sgRNA. Unfortunately, new transgenic lines expressing specific sgRNAs must be established de novo for each gene locus. The procedure is time consuming and significantly restricts the flexibility of the CRISPR/Cas9 system. Even with the sgRNA and Cas9 transgenic lines in hand, at least two or three rounds of hybridization are necessary to obtain homozygous or compound heterozygous mutants [5]. Moreover, additional rounds of hybridization are required to eliminate the sgRNA and Cas9 incorporated alleles, because the random insertion of these cassettes may disrupt the expression of endogenous genes and complicate subsequent research [8].

To overcome the drawbacks of the binary transgenic system, researchers have developed two DNA-free methods using Cas9 mRNA and Cas9 protein. Protocols using the Cas9 protein are more effective than those using Cas9 mRNA because the protein acts immediately following injection without a translational delay [9, 10]. In the Cas9 ribonucleoprotein (RNP)-based method, the T7 promoter is often chosen for in vitro transcription of the sgRNA [10]. However, the T7 promoter requires a GG dinucleotide at the RNA transcriptional start site for efficient transcription. A typical 20-nucleotide targeting region in the sgRNA is therefore generated with the structure 5′GGN_18_, and only genomic sites containing a GG dinucleotide at the corresponding position can be targeted by the Cas9 RNP. Although the 5′ GG rule can be relaxed to allow an adenine at either position [11], targetable genomic sequences are still limited. In addition, sgRNAs generated by T7 polymerase that initiate with 5′AG or 5′ GA exhibit poor mutagenic activity, due in part to 5′ end transcript heterogeneity [9].

To expand the number of targetable sites in the silkworm genome, we used the T7 promoter to add two supernumerary G residues to the 5′ end of a 20-nucleotide sgRNA targeting sequence. The GGN_20_ structure makes it possible to generate transcripts at high efficiency, and also provides a targeting sequence that can match a 20-nucleotide genomic target without a 5′ GG motif. However, the supernumerary residues may result in a 1- or 2-nucleotide mismatch immediately upstream of the perfectly matching genomic target. We therefore assessed the impact of the supernumerary 5′ GG residues on indel frequencies in vivo, and examined the transmission efficiencies for mutations generated using this system. Two well studied genes, *BmBLOS2* and *BmGR66*, were used as targets for proof-of-concept tests.

## Results

### Introduction of GG residues to the 5′ end of an sgRNA does not eliminate cleavage

To determine whether it is feasible to expand the range of sgRNAs synthesized in vitro using the T7 promoter, we designed sgRNAs containing two GG residues at their 5′ ends and used them to target genomic sequences that did or did not contain 5′ GG. Two sgRNA variations were tested. In the first case, the sgRNA target region had a GGN_18_ structure consisting of two GG residues and 18 additional nucleotides. GGN_18_ sgRNAs were used to target genomic sites that contained a perfect match to the sgRNA targeting sequence, including the two 5′ G residues. In the second case, the GG residues were added to a targeting sequence 20 nucleotides long, creating a GGN_20_ structure. We refer to the GG residues on GGN_20_ sgRNAs as “supernumerary”. GGN_20_ sgRNAs were tested at genomic sites that did not contain a 5′ GG sequence, but otherwise perfectly matched the 20 nucleotides in the target region.

Two genes were targeted to evaluate the performance of the GGN_18_ and GGN_20_ sgRNAs. *BmBLOS*2 is located on the Z chromosome, and is responsible for the biosynthesis of urate granules, which accumulate in epidermal cells and make the larval integument opaque. Because mutations in this gene result in an easily detected oily skin phenotype, *BmBLOS*2 is often used to confirm the efficacy of gene editing methods [1, 2, 4]. *GR66* is located on the third chromosome and has recently been identified as a key gustatory receptor responsible for the mulberry-specific feeding preference of the silkworm [7]. These two genes, for which null mutations generate detectable phenotypes, were used to test the efficacy of our strategy.

**Fig. 1:**
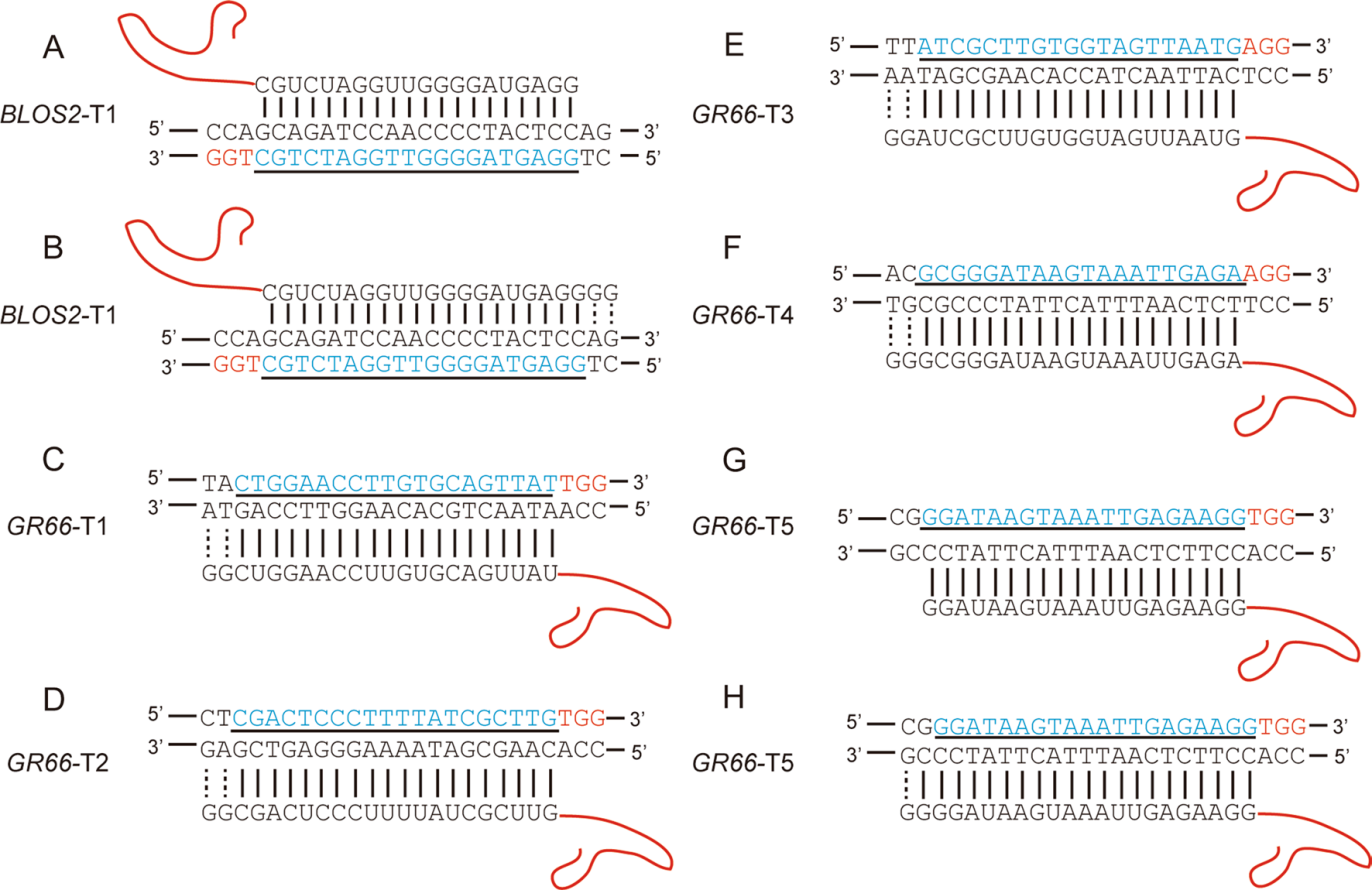
Genomic targets and corresponding sgRNAs in GGN_20_ or GGN_18_ formats. 20-nt genomic targets (not included the PAM) are shown in blue underlined text. The PAM is shown in red. The curved red line represents sequences that are common to all sgRNAs used in our experiments. Dotted lines represent mismatches between the sgRNA and genomic sequences. Note that GGN_18_ format sgRNAs were not generated for every target. **A** GGN_18_ format sgRNA for target *BLOS2*-T1. **B** GGN_20_ format sgRNA for target *BLOS2*-T1. **C** GGN_20_ format sgRNA for target *GR66*-T1 (no GGN_18_ format sgRNA). **D** GGN_20_ format sgRNA for target *GR66*-T2 (no GGN_18_ format sgRNA). **E** GGN_20_ format sgRNA for target *GR66*-T3 (no GGN_18_ format sgRNA). **F** GGN_20_ format sgRNA for target *GR66*-T4 (no GGN_18_ format sgRNA). **G** GGN_18_ format sgRNA for target *GR66*-T5. **H** GGN_20_ format sgRNA for target *GR66*-T5. Only one mismatch occurs between the 5′ end of this sgRNA and its genomic target

**Fig. 2:**
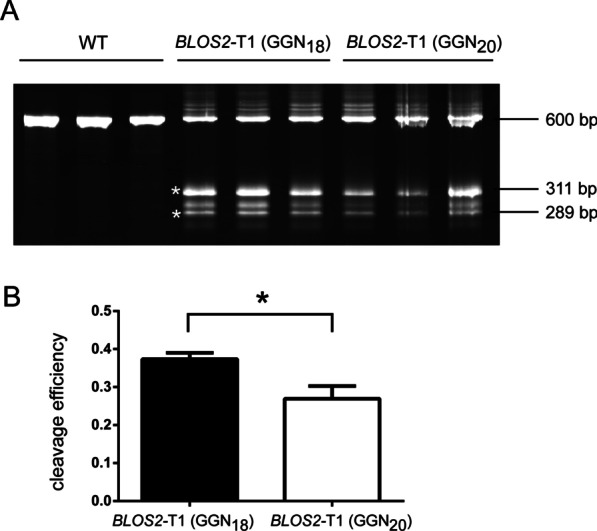
Cleavage efficiencies of GGN_18_- and GGN_20_-format sgRNAs targeting *BmBLOS2*. **A** *BLOS2-*T1 was targeted by sgRNAs in the GGN_18_ or GGN_20_-formats. A T7EN1 reaction was performed to evaluate cleavage efficiencies, and the products were separated by PAGE. The intact target is 600 bp in length, and the two bands derived from T7EN1 cutting are approximately 311 and 289 bp. The extra band that appears between 311 and 289 bp bands may be derived from SNPs in the amplified 600 bp region. The wildtype (WT) controls were amplified from *Dazao* genomic DNA by PCR. **B** Cleavage efficiencies were calculated by measuring band intensities. Bars represent mean values, and error bars represent SEM. *P*-values were determined using Student’s *t*-test. *P < 0.05. Please note that the uncropped original PAGE gel images are shown in Additional file 1: Figure S3

**Fig. 3:**
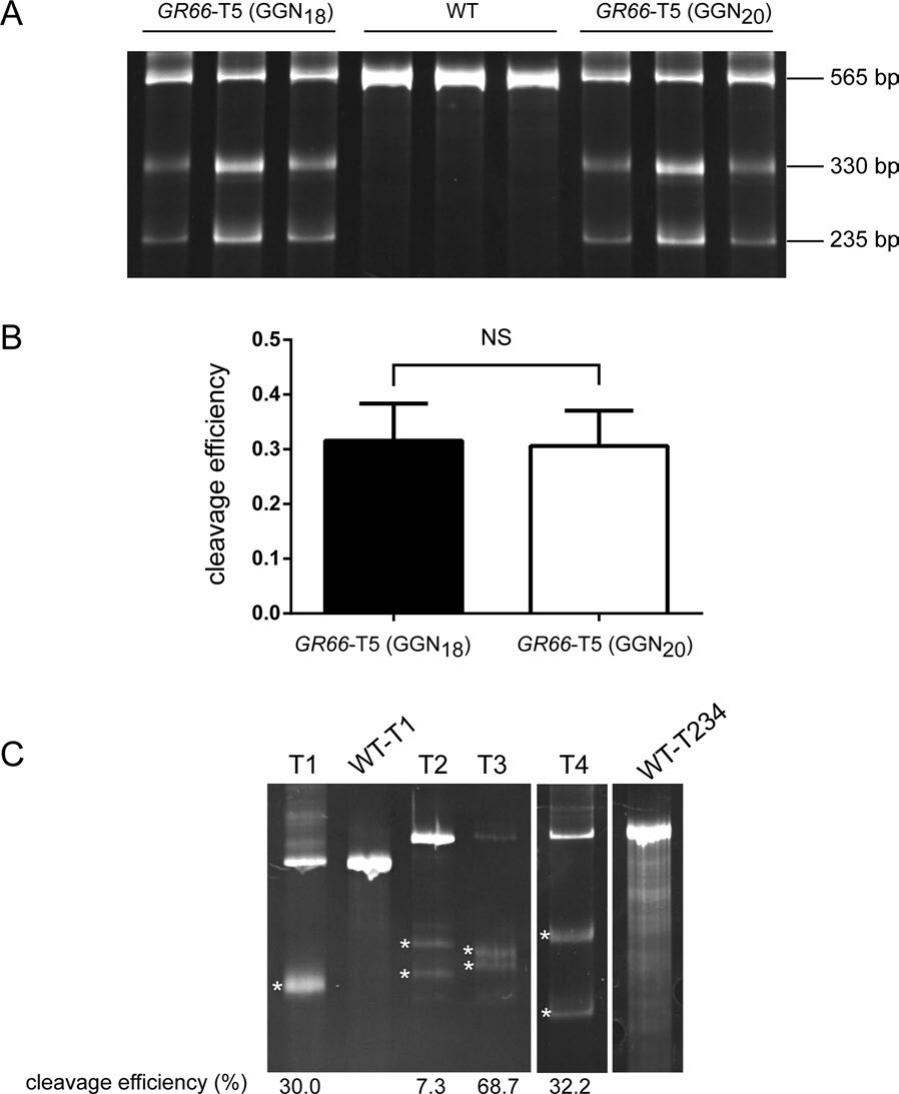
The influence of 5′ sgRNA supernumerary GG residues on cleavage efficiencies at targets with or without matching 5′ GG in *BmGR66*. **A** T7EN1 assay was performed after *GR66*-T5 was targeted by sgRNAs in the GGN_18_ and GGN_20_ formats. Cleavage products were separated by PAGE. Wildtype (WT) controls were amplified from *Dazao* genomic DNA by PCR. **B** Cleavage efficiencies were calculated by measuring band intensities. Bars represent mean values, and error bars represent SEM. *P*-values were determined using Student’s *t*-test. NS, not significant (*P* > 0.05). **C** A T7EN1 assay was performed to examine the cleavage efficiencies of sgRNAs targeting T1, T2, T3, and T4. WT-T1 (500 bp) was the uncleaved control for sgRNA *GR66*-T1, and WT-T234 (565 bp) was the uncleaved control for sgRNAs *GR66-*T2, T3, and T4. The two bands derived from T7EN1 digestion for sgRNA T1 were 248 bp and 252 bp; for sgRNA T2, 262 bp and 303 bp; for sgRNA T3, 274 bp and 291 bp; for sgRNA T4, 238 bp and 327 bp. Wildtype (WT) controls were amplified from *Dazao* genomic DNA by PCR. Cleavage efficiencies were calculated by measuring band intensities. Efficiency values are shown under each lane. Only one band was detected for sgRNA T1, because the two bands derived from T7EN1 cleavage are almost identical in size and were not resolved by the PAGE conditions used. Please note that the uncropped original PAGE gel images are shown in Additional file 1: Figure S3

Two targets were selected for initial tests in *BmBLOS2* and *BmGR66* (designated *BLOS2*-T1 and *GR66*-T5, respectively; Fig. 1). For both targets, sgRNAs in the GGN_18_ and GGN_20_ formats were synthesized using T7 RNA polymerase-mediated in vitro transcription. Note that the GGN_18_ sgRNAs match their respective targets in these genes perfectly (Fig. 1A, G), while the supernumerary GG residues in the GGN_20_ sgRNAs have two mismatches at *BLOS2*-T1 (5′ CT) and one mismatch at *GR66*-T5 (5′ CG) (Fig. 1B, H). Each sgRNA was separately mixed with Cas9 protein and incubated at room temperature to form a Cas9 protein/sgRNA complex, which was then injected into pre-blastoderm embryos. To examine indel frequencies, pools of 60 randomly selected injected embryos were harvested for genomic DNA extraction. Regions surrounding the sgRNA targets in *BmBLOS2* and *BmGR66* were PCR amplified and T7EN1 assays were performed to detect cleavage efficiencies. The results demonstrated that the GGN_18_ sgRNAs generated indels at higher efficiency (37.4% ± 1.6%) than the GGN_20_ sgRNAs (26.9% ± 3.3%) at the sgRNA *BLOS2*-T1 target (*P* < 0.05) (Fig. 2A, B). In contrast, no significant differences in indel frequencies were detected between GGN_18_ (31.6% ± 6.8%) and GGN_20_ (30.6% ± 6.5%) at *GR66-*T5 (*P* = 0.95) (Fig. 3A, B). The result at *GR66-*T5 suggests that a single 5′ G mismatch has a relatively minor effect on sgRNA cleavage efficiency compared with a 5′ GG mismatch.

To further test the method, we selected four additional genomic targets in *BmGR66,* none of which have native (G/A) (G/A) residues at their 5′ ends (*GR66*-T1, T2, T3, T4). Thus, at all four targets, sgRNAs in the GGN_20_ format will mismatch the genomic sequences at both supernumerary G residues (Fig. 1C–F). After in vitro synthesis from a T7 promoter, the sgRNAs were mixed with Cas9 protein, and the complexes were injected into pre-blastoderm silkworm embryos. DNA was extracted from pools of 60 randomly selected injected embryos for each sgRNA and subjected to a T7EN1 assay. *GR66*-T1, T2, T3, and T4 generated indels at efficiencies of 30.0%, 7.3%, 68.7%, and 32.2%, respectively (Fig. 3C). Importantly, the GGN_20_ sgRNA at *GR66*-T3 produced indels much more efficiently than did the GGN_18_ sgRNA at *GR66*-T5 (68.7% vs 31.6%). This result demonstrates that an sgRNA containing 5′ GG mismatches may outperform an sgRNA with 5′ GG matches within the same gene, suggesting that high knock-out efficiencies can sometimes be obtained without the requirement for 5′ GG matching.

In summary, the introduction of 5′ GG mismatches reduces the cutting efficiency of a GGN_20_ sgRNA, although a single 5′G mismatch has a relatively minor effect. Nevertheless, the presence of one or two 5′ mismatching G residues does not eliminate cleavage. Therefore, the addition of supernumerary 5′ GG residues via the T7 promoter permits the synthesis of sgRNAs at high efficiency and also makes it possible to expand the target repertoire of the Cas9 RNP-based method. Indeed, in some cases it is possible to achieve superior gene knockout efficiency at genomic targets that do not contain matching 5′ GG residues.

### Generation of somatic and heritable mutations in ***BmBLOS2*** using a GGN_20_ format sgRNA and Cas9

**Fig. 4:**
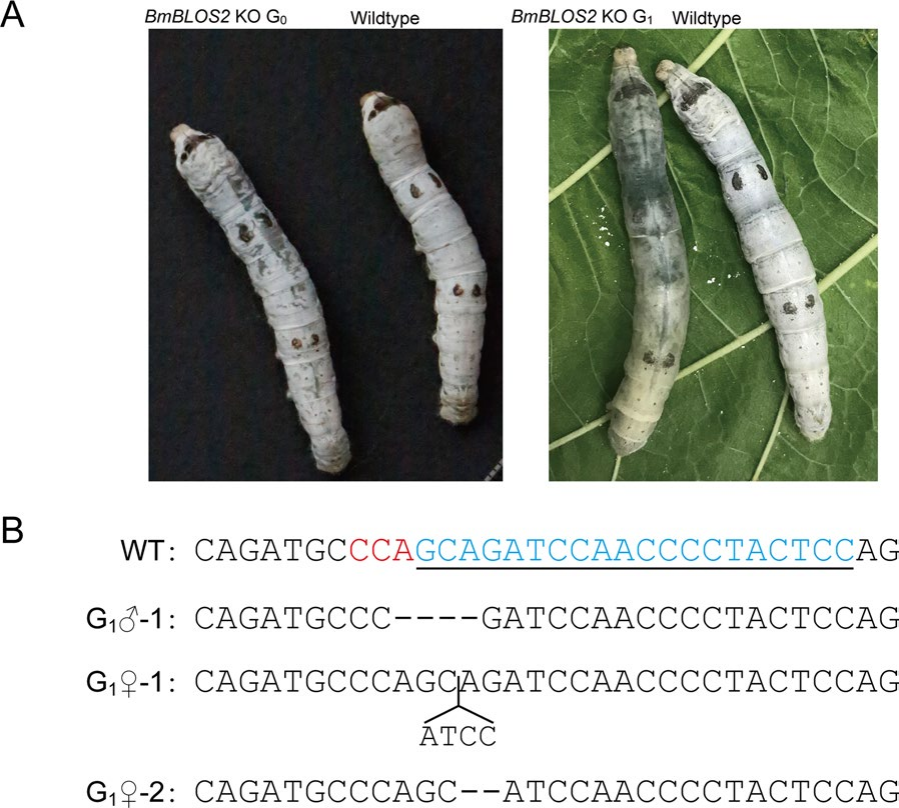
Generation of *BmBLOS2* knockout silkworms using GGN_20_ format sgRNA and detection of germline transmission of mutations. **A** Left panel: G_0_ *BmBLOS2* mutant silkworm exhibiting mosaic translucent epidermal phenotype. Right panel: G_1_ *BmBLOS2* null mutant silkworm exhibiting completely translucent epidermal phenotype. *Dazao* was used as the wildtype control. Note that gender cannot be determined by visual examination at the developmental stage shown here (fifth instar larva). KO, knockout. **B** Genotypes of G_1_ silkworms with detectable phenotypes. The wild type (WT) sgRNA target sequences are in blue and underlined, and PAM sequences are highlighted in red. In the mutants, dashes represent deleted nucleotides and the 4-nucleotide insertion is indicated using an open triangle

To test the germline transmission efficiency of mutations introduced by GGN_20_ sgRNAs, we injected 560 pre-blastoderm embryos with a complex containing the Cas9 protein and an sgRNA targeting *BmBLOS2* (*BLOS2*-T1). We detected a mosaic translucent epidermal phenotype in 42 fifth instar larvae out of a total of 81 in the injected generation (G_0_), yielding an efficiency of 51.9% (42/81) (Fig. 4A). G_0_ mosaic mutants with pronounced phenotypes were sib-mated. To assess heritability of the mutation, we randomly chose hatched larvae from three G_1_ egg batches (around 100 individuals per batch), and detected at least one larva in each batch with a completely translucent epidermal phenotype, indicative of homozygosity (Fig. 4A). A total of five larvae with completely translucent epidermal phenotypes were found in the three batches. One phenotypic larva from each batch (one male and two females) was chosen randomly to determine the genotype. The analysis showed that all three harbored mutations in the sgRNA target regions, and no wild type alleles were detected (Fig. 4B).

These results demonstrate that the transmission efficiency of mutations generated by GGN_20_ format sgRNAs is high enough to obtain phenotypic male (ZZ) homozygous mutants and female (ZW) hemizygous mutants in the *BmBLOS2* gene after only one breeding.

### Generation of heritable ***BmGR66*** mutants by co-injection of four sgRNAs with 5′ (G/A) (G/A) target mismatches

To examine the germline transmission efficiency of mutations introduced by multiple sgRNAs with 5′GG mismatches, we prepared GGN_20_ sgRNAs targeting T1, T2, T3, T4 in *BmGR66*, complexed them with Cas9 protein, and co-injected them into 80 preblastoderm embryos. Six embryos hatched, and genomic DNA was extracted from their wings after eclosion. Regions surrounding the four sgRNA targeting sites were amplified by PCR and then subcloned. Three to five randomly selected subclones generated from each silkworm were sequenced. Nucleotide substitutions, indels (small insertions or deletions), large fragment deletions, and inversions were detected surrounding all four sgRNA targeting sites (Additional file 1: Figure S1 and S2). No wildtype sequences were detected from any silkworm, and at least three mutant alleles were identified (Additional file 1: Figure S1 and S2), demonstrating that the silkworms were mosaic in the injected generation (G_0_). Similar outcomes have been reported previously in silkworms [4], zebrafish [12], and mouse [13, 14].

**Fig. 5:**
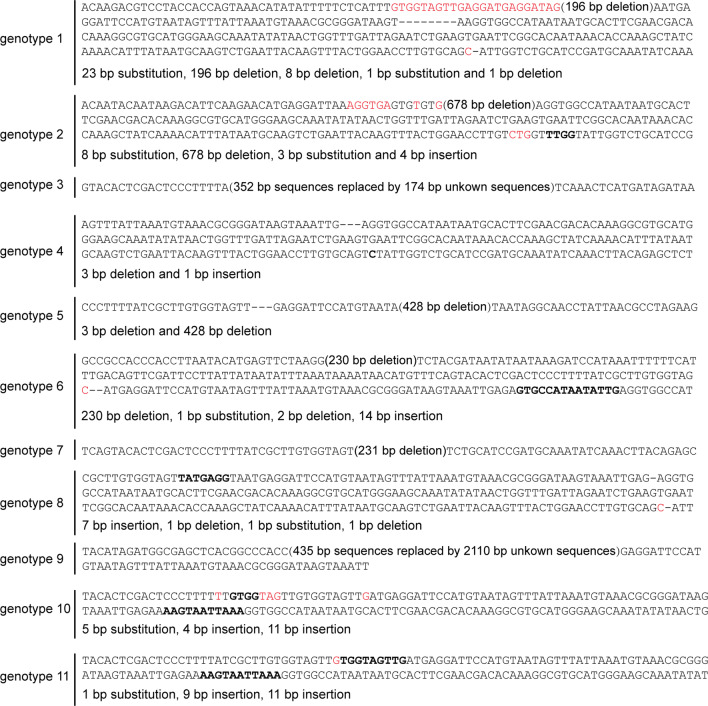
Genotypes recovered in G_1_ *BmGR66* knockout silkworms obtained by sib-mating of G_0_ mosaics. Mutations were detected in 17 G_1_ silkworms by PCR-amplification of regions surrounding the four sgRNA targeting sites. Amplified products were then subcloned and sequenced. Nucleotides shown in red are base substitutions; boldface represents insertions; dashes represent deletions. For large deletions or insertions, the sizes of the deleted or replaced regions are shown in parentheses

To test whether the mutations can be transmitted through the germline, we randomly selected one G_0_ male silkworm (#2) and one female G_0_ silkworm (#4), both with somatic mutations (Additional file 1: Figure S1 and S2). They were mated to obtain offspring, and newly moulted fifth-instar larvae in generation 1 (G_1_) were tested to determine their feeding preferences. 21 out of 79 ate cabbage leaves continuously, which are not normally consumed by wildtype silkworms, demonstrating an altered feeding preference (Additional file 2: Video S1). To correlate phenotype with genotype, 17 of the 21 phenotypic silkworms were randomly selected for analysis, and regions surrounding the sgRNA targets were amplified by PCR, subcloned, and sequenced. A total of 11 genotypes were recovered, and all harbored homozygous or compound heterozygous mutations. It is noteworthy that 7 of the 11 genotypes contained large (> 150 bp) insertions or deletions, which are readily obtained by simultaneous injection of multiple sgRNAs, and 10 of the 11 genotypes encoded truncated and presumably functionless proteins (Fig. 5 and Table 1). The changed feeding preferences resulting from the loss of *BmGR66* function are consistent with a previous report [7].

**Table 1:** Genotypes of 17 examined G_1_ *BmGR66* knockout silkworms

Silkworm ID	Genotype	
GR66 G_1_ ♀-1	Genotype 4	Homozygous
GR66 G_1_ ♀-2	Genotype 1 and 2	Compound heterozygous
GR66 G_1_ ♀-3	Genotype 4 and 2	Compound heterozygous
GR66 G_1_ ♀-4	Genotype 10 and 11	Compound heterozygous
GR66 G_1_ ♀-5	Genotype 4 and 2	Compound heterozygous
GR66 G_1_ ♀-7	Genotype 1 and 2	Compound heterozygous
GR66 G_1_ ♀-9	Genotype 4	Homozygous
GR66 G_1_ ♀-10	Genotype 3	Homozygous
GR66 G_1_ ♀-12	Genotype 2	Homozygous
GR66 G_1_ ♀-13	Genotype 9	Homozygous
GR66 G_1_ ♂-1	Genotype 6	Homozygous
GR66 G_1_ ♂-2	Genotype 5	Homozygous
GR66 G_1_ ♂-4	Genotype 1 and 2	Compound heterozygous
GR66 G_1_ ♂-5	Genotype 2	Homozygous
GR66 G_1_ ♂-6	Genotype 7	Homozygous
GR66 G_1_ ♂-8	Genotype 8	Homozygous
GR66 G_1_ ♂-9	Genotype 1	Homozygous

The corresponding genotypes are shown in detail in Fig. 5

Taken together, these results demonstrate that co-injection of multiple sgRNAs with mismatched 5′GG residues can efficiently generate somatic mutations at targeted regions in injected individuals. Furthermore, mutant alleles can be transmitted through the germline, and null mutants can be readily obtained by sib-mating G_0_ mosaics.

## Discussion

The delivery of CRISPR/Cas9 system into silkworms has been accomplished using the binary transgenic method [3, 5–7] and the Cas9 mRNA- or protein-based methods [4, 10]. In the binary method, the RNA polymerase type III promoter U6 is widely exploited to express sgRNA in vivo. The U6 promoter requires a guanosine nucleotide to initiate transcription, and thus genomic targets must contain a GN_19_NGG motif [15]. Strategies have been developed to circumvent this limitation. For example, a recent study demonstrated that replacing the U6 promoter with the H1 promoter expands the human genome target repertoire to include AN_19_NGG and GN_19_NGG [16]. In contrast, the silkworm U6 promoter effectively expresses sgRNAs initiated with any nucleotide [17], providing additional versatility. Unfortunately, even with this improvement, the transgene-based method is time consuming and difficult to implement [3, 5].

A significant advantage of the CRISPR/Cas9 system over ZFNs and TALENs is that mutagenesis can be directed to diverse genomic targets by simply exchanging the corresponding sgRNA, eliminating the labor that is required to reengineer the Cas9 enzyme [15, 18]. However, when the binary transgene-based method is used to deliver CRISPR/Cas9, every new genomic target requires a new transgenic line to express specific sgRNAs, limiting the flexibility of the system. Therefore, the DNA-free RNP-based CRISPR/Cas9 system has become increasingly popular in silkworms [10]. In this method, sgRNAs are synthesized in vitro, typically from a T7 promoter because of its high efficiency. The efficiency advantage of the T7 promoter is partially offset by its requirement for two guanosine nucleotides to initiate transcription, which significantly limits the number of targetable sites in the silkworm and other organisms. Although a Csy4-based gRNA cleavage strategy can be used to expand targetable regions for sgRNAs transcribed from a T7 promoter in vitro, it requires an additional step of purification and increases the risk of sgRNA degradation [19].

In this study, we developed a simple method to expand the target repertoire for sgRNAs in the Cas9 RNP system. Briefly, conventional sgRNAs are designed to contain a targeting sequence of 20 nucleotides to obtain efficient mutagenesis. When the T7 promoter is used to synthesize an sgRNA, the first two residues in the product are 5′GG, yielding a targeting sequence with the structure GGN_18_. Since the two G residues must match a corresponding 5′ GG motif at the genomic target, the range of possible targets is constrained. Because the 5′ GG residues perform unrelated roles in sgRNA synthesis and genomic targeting, we hypothesized that these functions could be satisfied independently with a slightly longer sgRNA structure. Specifically, we artificially introduced two supernumerary G residues to the 5′ end of a conventional 20-nt sgRNA, and then asked if the resulting GGN_20_ sgRNA generates mutations even if the supernumerary nucleotides have no corresponding matches in the genome. The remaining 20 nucleotides (N_20_) in the GGN_20_ sgRNA match the target perfectly. This strategy eliminates the need to select genomic targets containing 5′ GG residues, while preserving the ability to leverage the T7 promoter for efficient sgRNA synthesis.

GGN_20_ sgRNAs were tested against genomic targets that do not contain a native 5′ (G/A) (G/A) motif. We first examined the effect of the unpaired 5′ supernumerary guanines on mutagenesis efficiency in vivo in pooled injected embryos and individuals. As expected, the mutagenesis efficiencies of control sgRNAs that perfectly match their genomic targets (GGN_18_ format) equaled or surpassed those of sgRNAs containing two unpaired 5′ supernumerary guanines (GGN_20_ format), possibly because the GG mismatches at the 5′ end of sgRNA impair cleavage. These results are consistent with previous reports in zebrafish [20]. We also found that a single G mismatch at the 5′ end of the target has a relatively minor effect on cleavage efficiency. Nevertheless, the supernumerary guanines at the 5′ end do not abolish cleavage, which remains at levels high enough to generate somatic mutations and germline-transmissible mutations that can be recovered in succeeding generations. Homozygous or compound heterozygous mutants are readily obtained after only one breeding of mosaic G_0_ silkworms. Furthermore, we found that one GGN_20_ format sgRNA (*GR66*-T3) is much more efficient than the GGN_18_ format sgRNA *GR66*-T5 (68.7% vs 31.6%), demonstrating that sgRNAs with 5′ matched GG are not necessarily more efficient than sgRNAs with 5′ GG mismatches for targets within the same gene. Taken together, the results show that our improved method provides researchers with an expanded target repertoire, and can reach some targets at high efficiency even if matching 5′ GG residues are unavailable. Our method further increases the advantages of the Cas9 ribonucleoprotein (RNP)-based system over the widely used binary transgenic system [3, 5].

We also co-injected four different GGN_20_ sgRNAs into larvae and detected multiple mutant alleles in the G_0_ recipients. Homozygous and compound heterozygous G_1_ silkworms that harbored a wide range of mutations were easily generated. Almost all of the mutations (10 of 11) that were examined presumably encode truncated protein products, and 7 of the 11 contained large (> 150 bp) deletions or insertions. We hypothesize that the simultaneous activity of multiple sgRNAs not only increases the diversity of mutant alleles, but also favors the formation of large fragment insertions or deletions, in contrast to the smaller indels that are often generated when a single sgRNA is used. A previous study in zebrafish demonstrated that DNA repair machinery corrects double-stranded-breaks induced by Cas9 in a stereotypical and target-specific fashion, resulting in reduced mutant allele diversity [9]. Some somatic alleles were also over-represented in the germline, and at certain genomic targets the predominant alleles harboring indels were not frameshifts, making it difficult to obtain offspring with heritable null mutations [9]. Therefore, when the generation of null mutants is desired, injection of several different sgRNAs is much more effective than injection of a single sgRNA species. In addition, cleavage by multiple sgRNAs enhances indel formation efficiency and facilitates the production of complete gene knockouts in the injected generation [21, 22].

## Conclusions

In conclusion, we present a simple strategy to expand the targeting range of Cas9 RNP-based mutagenesis. The method leverages the highly efficient T7 promoter to produce sgRNAs with supernumerary 5′ GG residues that do not match corresponding nucleotides at the genomic target. Although the mismatches diminish the efficiency of mutagenesis, the efficiency is sufficiently high to generate somatic and heritable mutations. Homozygous and compound heterozygous mutants were readily obtained in a single generation by sib-breeding mosaics. The new method significantly expands the number of sites that can be targeted by the CRISPR/Cas9 system via the injection of in vitro transcribed sgRNAs in silkworm.

## Methods

### sgRNA design and synthesis

sgRNAs were designed using the CHOP-CHOP online utility (http://chopchop.cbu.uib.no/). sgRNA targeting sites are shown in Fig. 1. As described in a recent publication [23], the DNA template for T7 promoter used to drive in vitro transcription was constructed by PCR. Briefly, a customized oligonucleotide containing the T7 promoter and the sgRNA target sequence (N_20_ or N_18_) was designed as a forward primer with the sequence 5′-TAATACGACTCACTATAGG(N_20_ or N_18_)GTTTTAGAGCTAGAAATAGC. The T7 promoter sequences are underlined. The reverse primer was 5′-AAAAGCACCGACTCGGTGCCACTTTTTCAAGTTGATAACGGACTAGCCTTATTTTAACTTGCTATTTCTAGCTCTAAAAC-3′. sgRNA synthesis was performed using a RiboMax large scale RNA production system—T7 kit (Promega, cat. P1300), following the manufacturer’s instructions.

### Silkworm embryo microinjection

The bivoltine silkworm strain *Dazao* was obtained from the Silkworm Gene Bank of Southwest University (Chongqing, China) and was used in all experiments in this study. To collect non-diapaused eggs, silkworm eggs were incubated at 15˚C until hatching, and the larvae were reared at 25˚C and fed with fresh mulberry leaves until the wandering stage. Adult moths then oviposited non-diapaused eggs, which were used for microinjection. A mixture of sgRNA and Cas9 protein (Thermo fisher, cat. A36496) was incubated at room temperature for 15 min and microinjected into preblastoderm embryos within 5 h after oviposition. All silkworm embryo microinjection experiments in the study were conducted using this protocol.

Injected embryos were incubated at 25˚C and 80% humidity for 48 h. 60 silkworm eggs were then collected to extract genomic DNA for T7EN1 assays.

For experiments requiring hatched larvae, injected embryos were incubated at 25˚C and 80% humidity for approximately 11 days until hatching. Larvae were maintained at 25˚C and fed fresh mulberry leaves.

### T7EN1 assay and calculation of cleavage efficiency

Genomic DNA was extracted from pools containing 60 silkworm eggs, and PCR was performed to amplify sequences surrounding the sgRNA targeting site. To amplify the region surrounding the *GR66* T1 target, the primer was F: 5′-TCCCTTTTATCGCTTGTGGT-3′, and R: 5′-CTTCTAGGCGTTAATAGGTTGC-3′. To amplify the region surrounding the *GR66* T2, T3, T4 and T5 targets, the primer was F: 5′-TGATTCGGACTCACAAGACG-3′, and R: 5′-GGAAGAGAATGCGCCTGTAT-3′. To amplify the region surrounding the *BLOS2* T1 target, the primer was F: 5′-TGAGATGCTTTATGAGACAAGTCC-3′, and R: 5′-ATTTTCGAACCCGACAATGA-3′.

To determine sgRNA cleavage efficiencies, after the PCR products were annealed, they were subjected to T7EN1 (NEB, cat. M0302S) digestion at 37 °C for 1 h, and fragments were then separated by polyacrylamide gel electrophoresis (PAGE). The detailed protocol is available in our previous publication [24]. Band intensities were measured after PAGE using Image J [25]. Using a previously described approach [26], cleavage efficiencies were calculated by the formula 100 × (1 − (1 − (*b* + *c*)/(*a* + *b* + *c*))1/2), where *a* is the integrated intensity of the undigested PCR product, and *b* and *c* are the integrated intensities of each cleavage product.

### Genotype analysis

Genomic DNA was extracted from moth wings, and regions surrounding the sgRNA targeting sites were amplified by PCR. The PCR primers for genotyping *BmBLOS2* were F: 5′-TGAGATGCTTTATGAGACAAGTCC-3′, and R: 5′-ATTTTCGAACCCGACAATGA-3′. The PCR primers for genotyping *BmGR66* were F: 5′-CCCCATCCTTCAAACTGAAA-3′, and R: 5′-ACATTTGTTCAACCCCAAGC-3′. PCR products were subcloned into pEASY-blunt-zero vectors (TransGen, cat. CB501-01) and sequenced.

### Statistical analysis

All values are presented as means ± SEM. Student’s *t*-test was used to compare means. Differences were defined to be statistically significant at *P* < 0.05.

## Supplementary Information

**Additional file 1: Figure S1**. Genotypes of three G_0_*BmGR66* knockout silkworms generated by co-injection of sgRNAs T1, T2, T3, and T4 complexed with Cas9 protein. **Figure S2**. Genotypes of two G_0_ *BmGR66* knockout silkworms generated by co-injection of sgRNA T1, T2, T3 and T4 with Cas9 protein. **Figure S3**. Uncropped original PAGE gel image.

**Additional file 2. Video S1**. A compound heterozygous mutant of *BmGR66* eating cabbage leaves.

## Data Availability

All data generated or analysed during this study are included in this published article and its supplementary information files.

## Abbreviations

CRISPR/Cas9: Clustered regularly interspersed short palindromic repeat/CRISPR-associated protein 9
gRNA: Guide RNA
PAM: Protospacer adjacent motif
WT: Wildtype
KO: Knockout
RNP: Ribonucleoprotein
ZFNs: Zinc-finger nucleases
TALENs: Transcription activator-like effector nucleases
