# Supplementary material for: Expansion of targetable sites for the ribonucleoprotein-based CRISPR/Cas9 system in the silkworm Bombyx mori
Source: BMC Biotechnol. 2021 Sep 20;21:54. doi: 10.1186/s12896-021-00714-6 (PMC8454041; doi:10.1186/s12896-021-00714-6)
Supplement: Supplementary file 1 — Additional file 1: Figure S1. Genotypes of three G0BmGR66 knockout silkworms generated by co-injection of sgRNAs T1, T2, T3, and T4 complexed with Cas9 protein. Figure S2. Genotypes of two G0 BmGR66 knockout silkworms generated by co-injection of sgRNA T1, T2, T3 and T4 with Cas9 protein. Figure S3. Uncropped original PAGE gel image. [file 12896_2021_714_MOESM1_ESM.docx]

**Expansion of targetable sites for the ribonucleoprotein-based CRISPR/Cas9 system in the silkworm** ***Bombyx mori***

Yun-long Zou^1^, Ai-jun Ye^1^, Shuo Liu^1^, Wen-tao Wu^1^, Li-feng Xu^1^, Fang-yin Dai^1^, Xiao-ling Tong^1,^*

1, State Key Laboratory of Silkworm Genome Biology; Key Laboratory of Sericultural Biology and Genetic Breeding, Ministry of Agriculture and Rural Affairs; College of Sericulture, Textile and Biomass Sciences; Southwest University, Chongqing 400715, P. R. China

*Correspondence: Xiao-ling Tong

Email address: xltong@swu.edu.cn

Telephone：+86-23-68250551


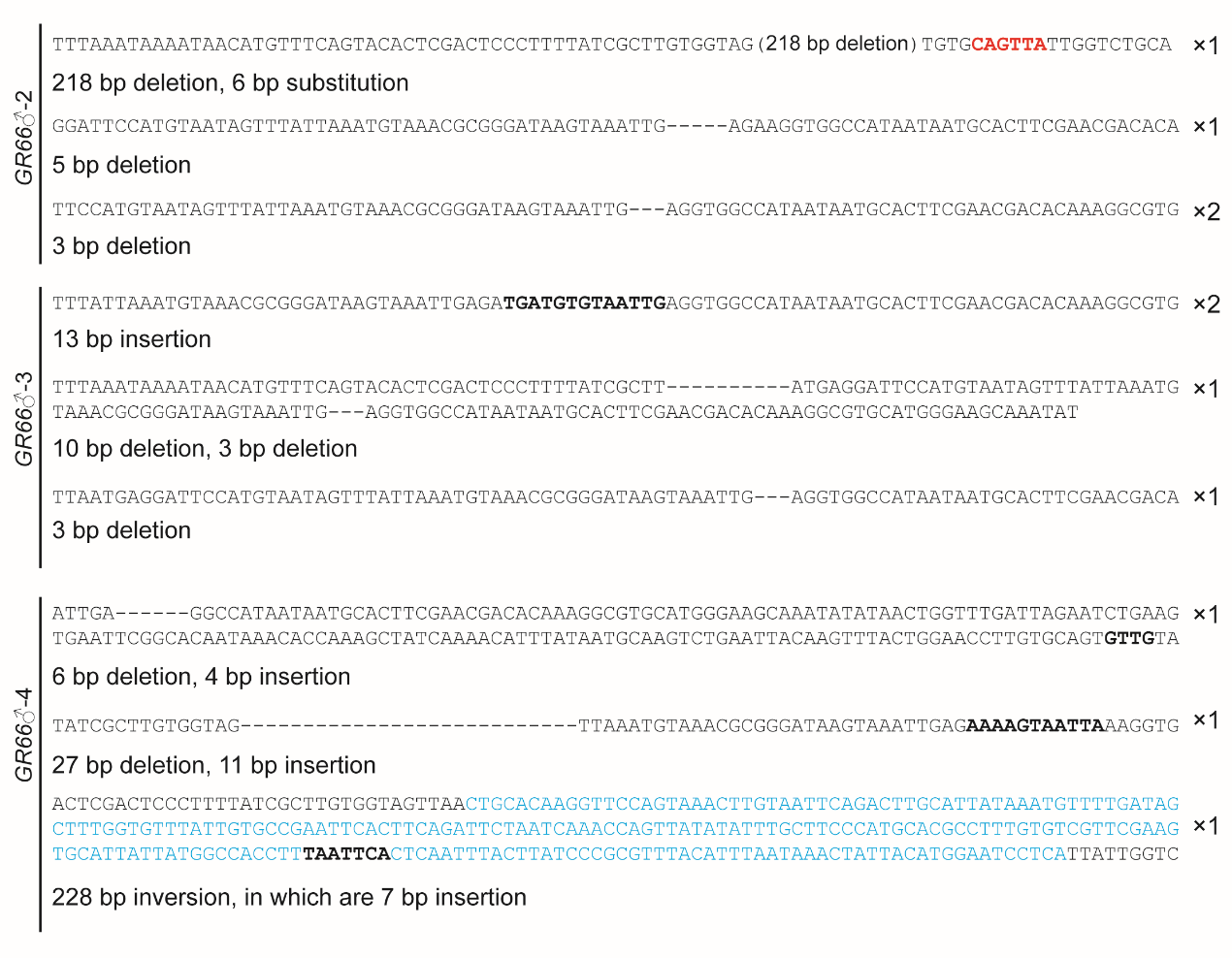


**Figure S1.** **Genotypes of three G_0_ *BmGR66* knockout silkworms generated by co-injection of sgRNAs T1, T2, T3, and T4 complexed with Cas9 protein**

Mutations were detected in three male G_0_ silkworms by PCR-amplification of regions surrounding the four sgRNA targeting sites. Amplified products were then subcloned and sequenced. Nucleotides shown in red are base substitutions; boldface represents insertions; dashes represent deletions; blue indicates an inversion. For large deletions, the size of the deleted region is shown in parentheses. The numbers of specific mutations detected in subclone sequencing are shown to the right of each sequence.


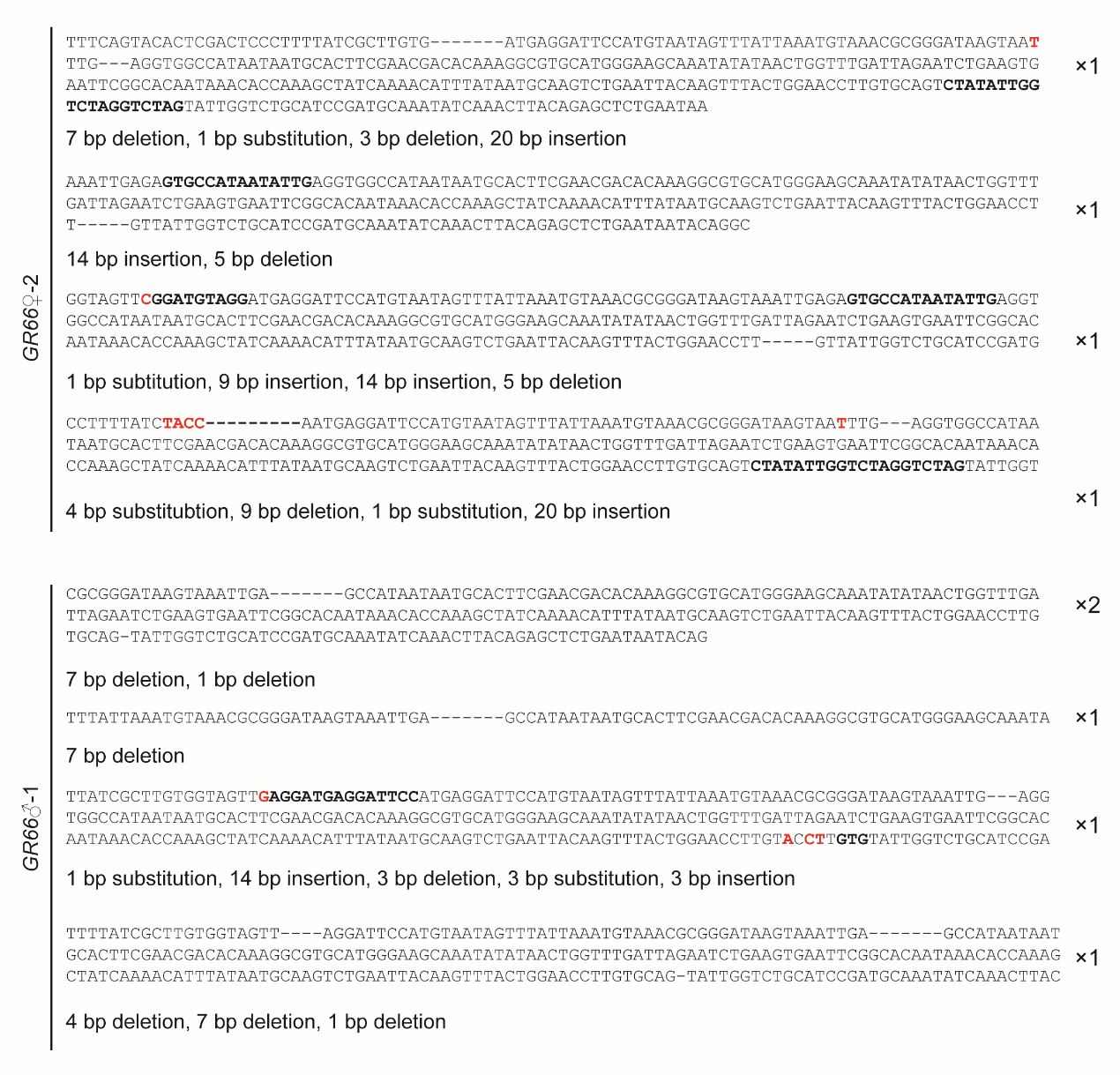


**Figure S2. Genotypes of two G_0_ *BmGR66* knockout silkworms generated by co-injection of sgRNA T1, T2, T3 and T4 with Cas9 protein**

Mutations were detected in one male G_0_ and one female G_0_ silkworm by PCR-amplification of regions surrounding the four sgRNA targeting sites. Amplified products were then subcloned and sequenced. Nucleotides shown in red are base substitutions; boldface represents insertions; dashes represent deletions. The numbers of specific mutations detected in subclone sequencing are shown to the right of each sequence.


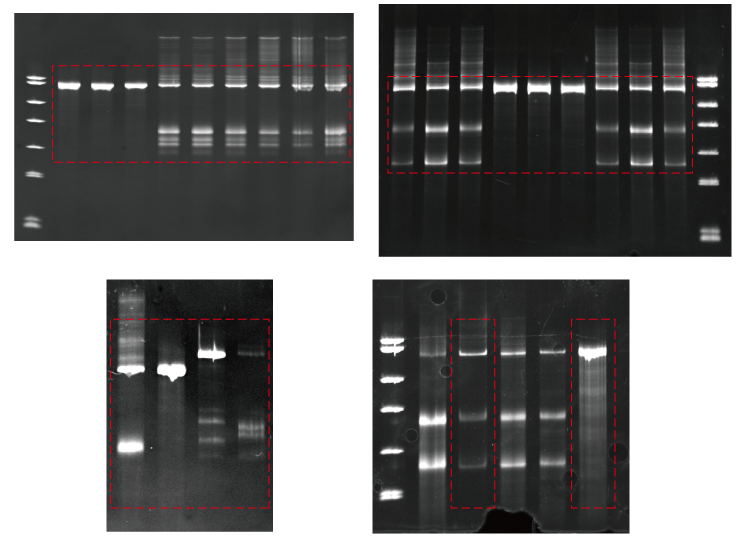


**Figure S3**. **Uncropped original PAGE gel image.** The cropped area is indicated by a red dashed rectangle.


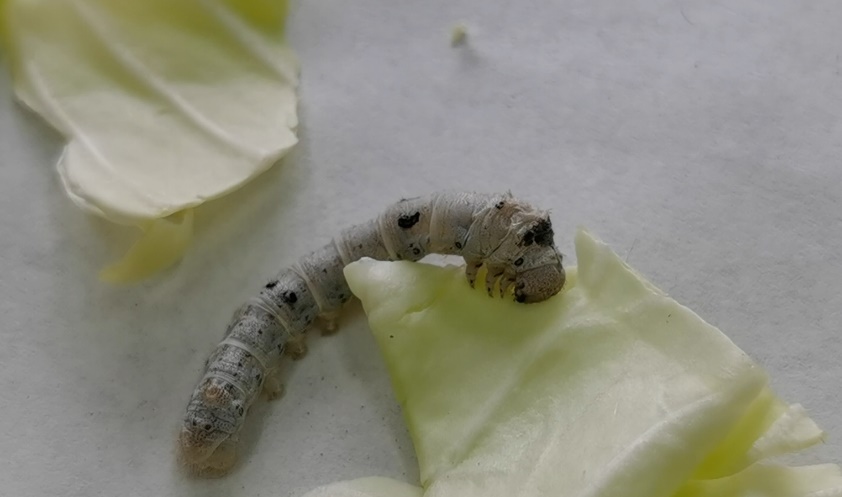


**Video S1. A compound heterozygous mutant of *BmGR66* eating cabbage leaves.**
